# Assessment of Functional Characteristics of Amnestic Mild Cognitive Impairment and Alzheimer's Disease Using Various Methods of Resting-State FMRI Analysis

**DOI:** 10.1155/2015/907464

**Published:** 2015-06-09

**Authors:** Jungho Cha, Jung-Min Hwang, Hang Joon Jo, Sang Won Seo, Duk L. Na, Jong-Min Lee

**Affiliations:** ^1^Department of Biomedical Engineering, Hanyang University, Seoul 133-791, Republic of Korea; ^2^Section on Functional Imaging Methods, Laboratory of Brain and Cognition, National Institute of Mental Health, National Institutes of Health, Bethesda, MD 20892, USA; ^3^Department of Neurology, Samsung Medical Center, Sungkyunkwan University School of Medicine, Seoul 135-710, Republic of Korea

## Abstract

Resting-state functional magnetic resonance imaging (RS FMRI) has been widely used to analyze functional alterations in amnestic mild cognitive impairment (aMCI) and Alzheimer's disease (AD) patients. Although many clinical studies of aMCI and AD patients using RS FMRI have been undertaken, conducting a meta-analysis has not been easy because of seed selection bias by the investigators. The purpose of our study was to investigate the functional differences in aMCI and AD patients compared with healthy subjects in a meta-analysis. Thus, a multimethod approach using regional homogeneity, amplitude of low-frequency fluctuation (ALFF), fractional ALFF (fALFF), and global brain connectivity was used to investigate differences between three groups based on previously published data. According to the choice of RS FMRI approach used, the patterns of functional alteration were slightly different. Nevertheless, patients with aMCI and AD displayed consistently decreased functional characteristics with all approaches. All approaches showed that the functional characteristics in the left parahippocampal gyrus were decreased in AD patients compared with healthy subjects. Although some regions were slightly different according to the different RS FMRI approaches, patients with aMCI and AD showed a consistent pattern of decreased functional characteristics with all approaches.

## 1. Introduction

Resting-state functional magnetic resonance imaging (RS FMRI) does not require subjects to perform a specific task or stimuli to be applied; it simply requires the participants to keep their mind clear. Not having to perform a task provides a significant benefit, especially for patients who may have difficulties performing such a task. As a result, RS FMRI has been widely used to analyze the functional differences in Alzheimer's disease (AD) and amnestic mild cognitive impairment (aMCI) patients compared with healthy subjects. Although many clinical studies of aMCI and AD patients using RS FMRI have been undertaken, conducting a meta-analysis has not been easy. One limitation has been the use of a seed-based approach. Typically, seeds are based on an anatomical atlas, using either the location of activity during the task or the standardized coordinates. The choice of seed may include selection bias by the investigator, and the patterns of functional connections may be quite different depending on the seed location [1]. Therefore, studies that used a seed-based approach are not suitable for inclusion in a meta-analysis. Apart from the seed-based approach, other approaches have been used to analyze the findings from RS FMRI. To avoid selection bias, several methods such as regional homogeneity (ReHo), amplitude of low-frequency fluctuation (ALFF), fractional ALFF (fALFF), and global brain connectivity (GBC) can be considered for meta-analysis.

ReHo is based on the similarity of a given voxel to its neighbor voxels over a time series [2]. The similarity over a number of time series can be measured using Kendall's coefficient concordance (KCC) [3]. This method is based on the hypothesis that significant brain activities are more likely to occur in clusters rather than in a single voxel [4]. The patterns identified using ReHo were found to be similar to those in regions deactivated during demanding cognitive tasks in previous positron emission tomography studies [5, 6]. This indicates that the ReHo method can be used to investigate the complexity of human brain function. In addition, previous studies have shown that the ReHo index patterns in the resting state can be used as a potential clinical marker for aMCI and AD [7, 8].

The ALFF and fALFF methods measure regional spontaneous brain activity. The ALFF technique measures the amplitude of resting-state spontaneous brain activity by calculating the square root of the power spectrum in the low-frequency range [9]. However, the ALFF is weak because of physiological noise near the large ventricles [10, 11]. To overcome these problems, previous studies have suggested use of the fALFF method [10]. The fALFF method measures the ratio of the low-frequency power spectrum to that of the entire frequency range and has been shown to have improved sensitivity and specificity in the detection of spontaneous brain activity compared with the ALFF approach [10, 11]. Previous studies have shown that the specific patterns of ALFF and fALFF in aMCI and AD patients provide insights into the biological mechanisms of the disease [12–15].

The technique of GBC identifies the brain's most globally connected regions. GBC uses the seed-based correlations of each voxel with all other brain voxels [16]. These values are then averaged together. The high-GBC regions occur mainly in the cognitive control network (CCN) and the default mode network (DMN) [17]. Therefore, the GBC patterns represent the complex brain functions. A previous study showed that the GBC patterns could explain the patterns of vulnerability seen in AD patients [18]. However, the GBC patterns of aMCI patients were clearly understood.

The purpose of our study was to investigate the regions of functional differences in aMCI and AD patients compared with healthy aging subjects using a meta-analysis. Thus, a multimethod approach using ReHo, ALFF/fALFF, and GBC was used to investigate differences between three groups. To aid this meta-analysis, we analyzed existing data published on resting-state FMRI [19]. In previous study using spatial independent component analysis (sICA), we showed that there were significant differences between healthy subjects and patients with aMCI and AD. The results of several approaches using the same data can considerably encourage the further meta-analysis.

## 2. Materials and Methods

### 2.1. Subjects

This study reanalyzed previously published RS FMRI data [19]. Sixty-two healthy subjects (male/female ratio, 17/45; age, 68.5 ± 8.0), 34 patients with aMCI (18/16, 68.4 ± 7.9 years old), and 37 patients with AD (10/27, 72.8 ± 8.2 years old) participated in this study. We obtained written informed consent, according to the Declaration of Helsinki, from all subjects and the study was approved by the Institutional Review Board of the Samsung Medical Center, Seoul, South Korea. The demographic and clinical data of the participants are presented in Table 1.

### 2.2. Data Acquisition

All imaging was carried out at the Samsung Medical Center. The scanner was a Philips Intera Achieva 3.0 Tesla scanner equipped with an 8-channel SENSE head coil (Philips Healthcare, The Netherlands). A high-resolution T1-weighted anatomical image was acquired using a magnetization-prepared gradient echo (MPRAGE) sequence (TR = 9.9 ms; TE = 4.6 ms; flip angle = 8°; 0.5 × 0.5 × 0.5 mm^3^ voxel resolution). And whole-brain echo-planar imaging (EPI) time-series scans (TR = 3 s; TE = 35 ms; flip angle = 90°; 1.7 × 1.7 × 4 mm^3^ voxel resolution) were acquired. RS FMRI data consisted of 100 volumes. During each scan, participants were instructed to rest with their eyes open.

### 2.3. Preprocessing of RS FMRI Data

Preprocessing of the RS FMRI data was performed using Analysis of Functional NeuroImage (AFNI) software (http://afni.nimh.nih.gov/) [20]. To correct for physiological noise, we first identified cardiac and respiratory noises of the RS FMRI data [21] using PESTICA software (Physiologic EStimation by Temporal ICA, http://www.nitrc.org/projects/pestica/). PESTICA includes IRF-RETROICOR, an improved correction method [22] that calculates the impulse response function (IRF) of each heartbeat or breath. For stabilization of the magnetic field and signal equilibrium, the initial three volumes from each functional image were removed. Slice timing and head motion corrections at the RS echo-planer imaging (EPI) time courses were then applied. Then, data were corrected using the anatomy-based correlation corrections (ANATICOR) method [23]. The data that were regressed included (1) six parameters obtained from the correction of head motion, (2) the signal from the eroded large ventricle mask, and (3) the signal from a region of the local white matter erosion mask (*r* = 15 mm). To obtain the large ventricle masks and white matter mask, T1 images registered and corrected for intensity non-uniformities resulting from inhomogeneity in the magnetic field were divided into white matter, gray matter (GM), cerebrospinal fluid, and background using an advanced neural-net classifier [24]. Although there has been debate about global signal, we did not perform the regression analysis with global signal. Previous studies showed that global signal regression may induce artificial negative correlations and influence the long- and short-range functional connections [25–27]. The anatomical T1 image was registered to the functional images using the local Pearson correlation cost function [28], and all masks were converted to EPI space. The large ventricle mask and the white matter mask were eroded by one voxel to minimize partial volume effects.

### 2.4. Postprocessing for Several Methods

#### 2.4.1. ALFF/fALFF Analysis

We used the AFNI software to process the ALFF data, which have been depicted in previous studies [9, 29]. The time series were first converted to the frequency domain using a fast Fourier transform (FFT), and the power spectrum was then acquired. As the transformed frequency within the power spectrum is proportional to the square of the amplitude of this frequency component in the original time series, the power spectrum obtained by FFT was calculated and averaged across the frequency range 0.009–0.08 Hz at each voxel over the time courses. This averaged square root was taken as the ALFF [9]. To improve the ALFF approach, we also used the fALFF, the ratio of the power of the low-frequency fluctuations to that of the entire frequency range (0.009–0.25 Hz), which has been reported to be more sensitive than the original ALFF in detecting spontaneous brain activity [10]. After the calculation of the ALFF and fALFF maps, the GM mask was applied to reduce the inclusion of unwanted blood oxygen level-dependent signals or other physiological signals that occur because of large draining vessels. The ALFF and fALFF maps then underwent spatial smoothing with a 6 mm full-width-at-half-maximum (FWHM) Gaussian kernel and were normalized to the MNI152 template.

#### 2.4.2. Regional Homogeneity Analysis

Regional homogeneity was calculated by the KCC values using the AFNI software. This method has been described as measuring the similarity of the time series within a cluster defined by the nearest neighbor voxels (27, 19, or 7, including a given voxel) in the whole brain [2]. Before the calculation of regional homogeneity, band-pass filtering (0.009 Hz < *f* < 0.08 Hz) was performed and the GM mask was applied. And the images underwent spatial smoothing with a 6 mm FWHM Gaussian kernel and were normalized to the MNI152 template. Then, the KCC was computed using

(1) $$\begin{array}{c} W=\frac{\sum {\left(R_{i}\right)}^{2}-n{\left(\overset{-}{R}\right)}^{2}}{\left(1/12\right)K^{2}\left(n^{3}-n\right)}, \end{array}$$

where $W\left[\begin{array}{cc} 0 & 1 \end{array}\right]$ is the value of KCC for a given set of voxels, *R*_*i*_ is the sum rank of the *i*_th_ time point, $\overset{-}{R}=(\left(n+1\right)\times K)/2$ is the mean of *R*_*i*_, *K* is the number of time courses within a measured cluster, and *n* is the number of ranks. We set the number of neighboring voxels to 27. The individual ReHo maps were obtained by computing KCC for each voxel.

#### 2.4.3. Global Brain Connectivity (GBC) Analysis

GBC analysis [17] was calculated by globally connected regions. Before the calculation of the GBC maps, preprocessed functional images were performed band-pass filtered (0.009 Hz < *f* < 0.08 Hz), GM masked, spatial smoothed with 6 mm FWHM Gaussian kernel and normalized to the MNI152 template. The GBC maps, which calculated the correlation coefficients with all the other voxels within brain for each voxel, were computed with AFNI software (3dTcorrMap). The correlation values were converted to *z* values using Fisher's *z* transformation. The transformed values were averaged and the value was assigned to that voxel. The individual GBC map was then obtained.

### 2.5. Group Comparisons

All the results from the different RS FMRI techniques were masked out, with the group mask obtained by selecting a threshold of 0.3 on the mean GM map of all subjects. To explore differences in the functional characteristics between the three groups, an analysis of covariance (ANCOVA) was performed using sex, age, and education as covariates. The correction of Type I errors (parameters: individual voxel *P* value = 0.01, simulated 10,000 times iteratively, 6 mm FWHM Gaussian filter width with the group mask) was reckoned using Monte Carlo simulations with AFNI's AlphaSim software program. The AlphaSim program provides an overall significance level achieved for various combinations of cluster size thresholds and probability thresholds for each voxel [30]. This is performed by Monte Carlo simulation of the process of image generation, masking, spatial correlation of voxels, voxel intensity thresholding, and cluster identification. The probability of the false positive detection per image is determined from the frequency count of cluster size [31].

The significance level was set at *P*_*α*_ < 0.05 (uncorrected individual voxel height threshold of *P* < 0.01, *F* > 4.776) and a cluster size of 864 mm^3^.* Post hoc *two-sample *t*-tests were employed between pairs of groups for voxelwise statistics at a corrected significance level of *P*_*α*_ < 0.05.

## 3. Results

**Figure 1:**
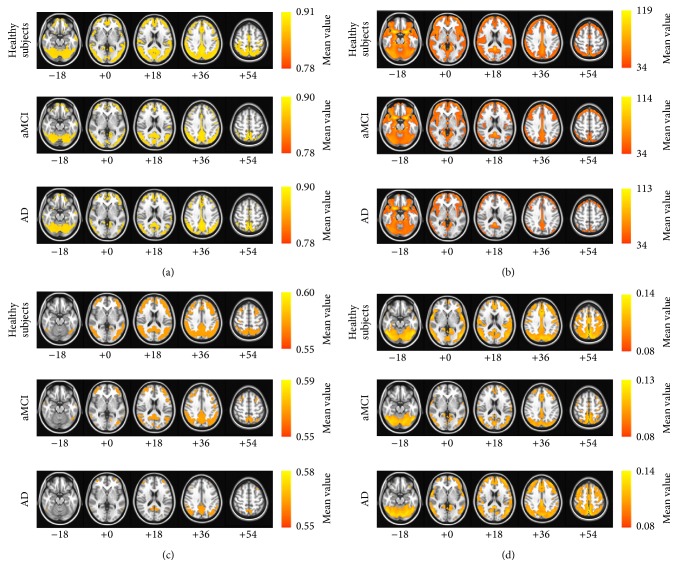
The mean images of each resting-state FMRI analysis approach: (a) regional homogeneity (ReHo), (b) amplitude of low-frequency fluctuation (ALFF), (c) fractional ALFF, and (d) global brain connectivity (GBC). The first row of each approach is the map for the healthy subjects, the second row of each approach is the map for the patients with aMCI, and the third row of each approach is the map for the patients with AD. The images are oriented with the anterior side placed at the top and the left side placed to the right. The red and blue colors represent positive and negative functional connectivity, respectively.

To allow visual inspection of the different approaches, mean images were generated for each group. The majority of the clusters were consistent across all groups and the patterns were quite similar to the previous results for each approach (Figure 1). These regions included the PCC/precuneus, middle frontal gyrus, anterior cingulate cortex (ACC), inferior parietal lobule, and middle temporal gyrus.

**Figure 2:**
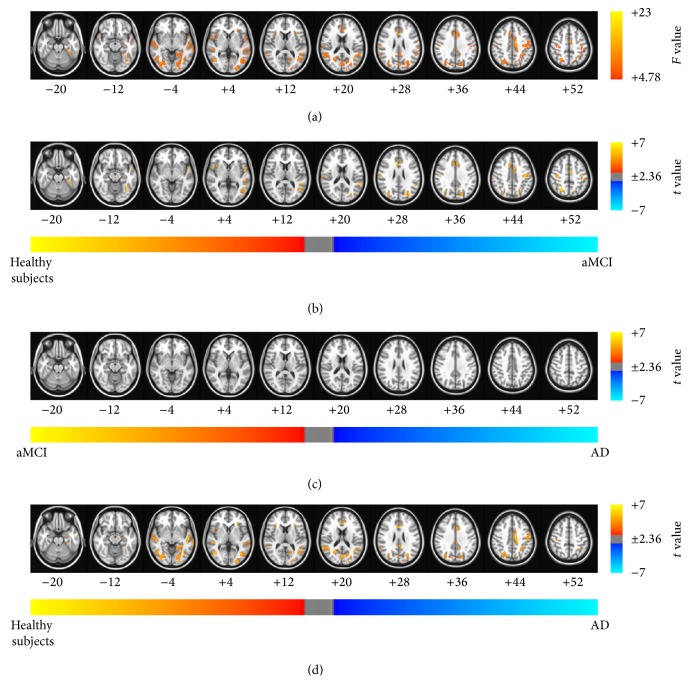
Brain regions exhibiting significant differences in the regional homogeneity (ReHo) index. (a) Brain regions showed significant differences in ReHo between healthy subjects and patients with aMCI and patients with AD (*P*_*α*_ < 0.05 (uncorrected *P* < 0.01, *F* > 4.78, 864 mm^3^, and AlphaSim corrected)). The results of the* post hoc* two-sample *t*-tests between pairs of the healthy subjects and patients with AD and patients with aMCI were as follows: significant differences in brain regions were found (b) in patients with aMCI compared with healthy subjects, (c) in patients with AD compared with patients with aMCI, and (d) in patients with AD compared with healthy subjects (*P*_*α*_ < 0.05). The images are oriented with the anterior side placed at the top and the left side placed to the right.

**Figure 3:**
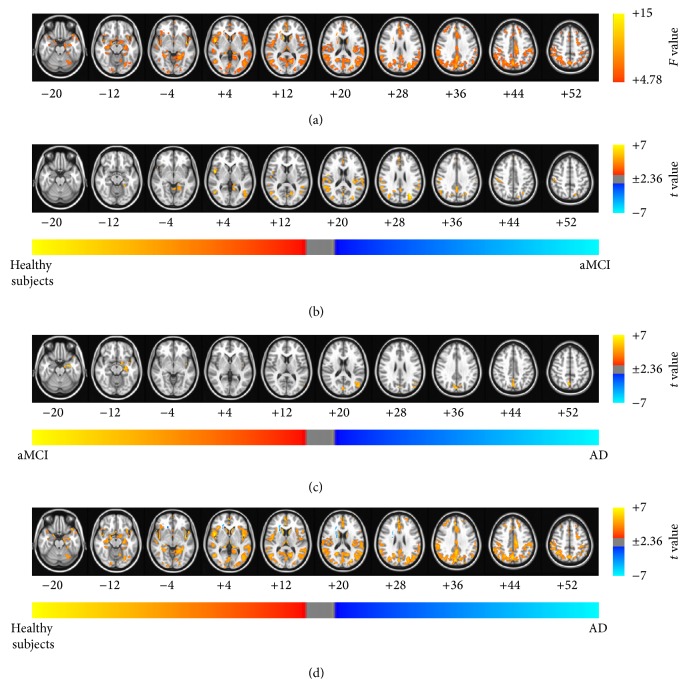
Brain regions exhibiting significant differences in the amplitude of low-frequency fluctuation (ALFF) index. (a) Brain regions showed significant differences in ALFF between healthy subjects and patients with aMCI and patients with AD (*P*_*α*_ < 0.05 (uncorrected *P* < 0.01, *F* > 4.78, 864 mm^3^, and AlphaSim corrected)). The results of the* post hoc* two-sample *t*-tests between pairs of the healthy subjects and patients with AD and patients with aMCI were as follows: significant differences in brain regions were found (b) in patients with aMCI compared with healthy subjects, (c) in patients with AD compared with patients with aMCI, and (d) in patients with AD compared with healthy subjects (*P*_*α*_ < 0.05). The images are oriented with the anterior side placed at the top and the left side placed to the right.

**Figure 4:**
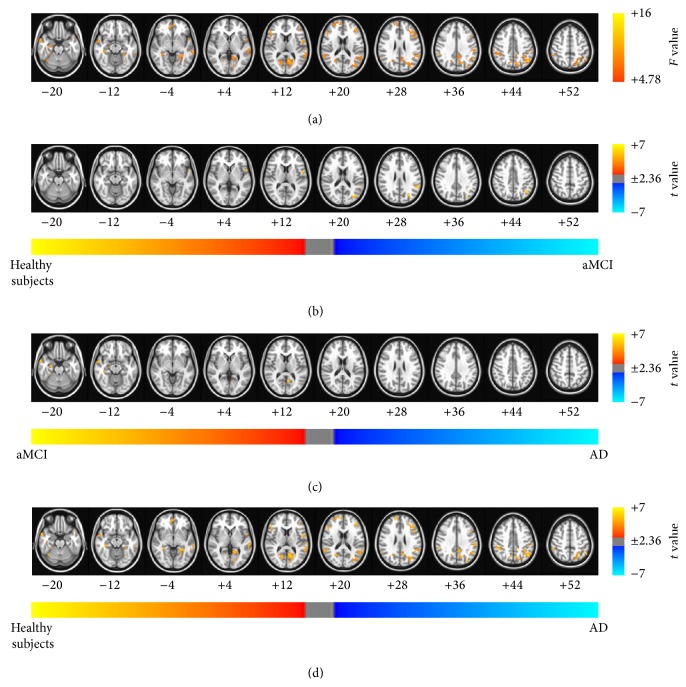
Brain regions exhibiting significant differences in the fractional ALFF (fALFF) index. (a) Brain regions showed significant differences in fALFF between healthy subjects and patients with aMCI and patients with AD (*P*_*α*_ < 0.05 (uncorrected *P* < 0.01, *F* > 4.78, 864 mm^3^, and AlphaSim corrected)). The results of the* post hoc* two-sample *t*-tests between pairs of the healthy subjects and patients with AD and aMCI were as follows: significant differences in brain regions was found (b) in patients with aMCI compared with healthy subjects, (c) in patients with AD compared with patients with aMCI, and (d) in patients with AD compared with healthy subjects (*P*_*α*_ < 0.05). The images are oriented with the anterior side placed at the top and the left side placed to the right.

**Figure 5:**
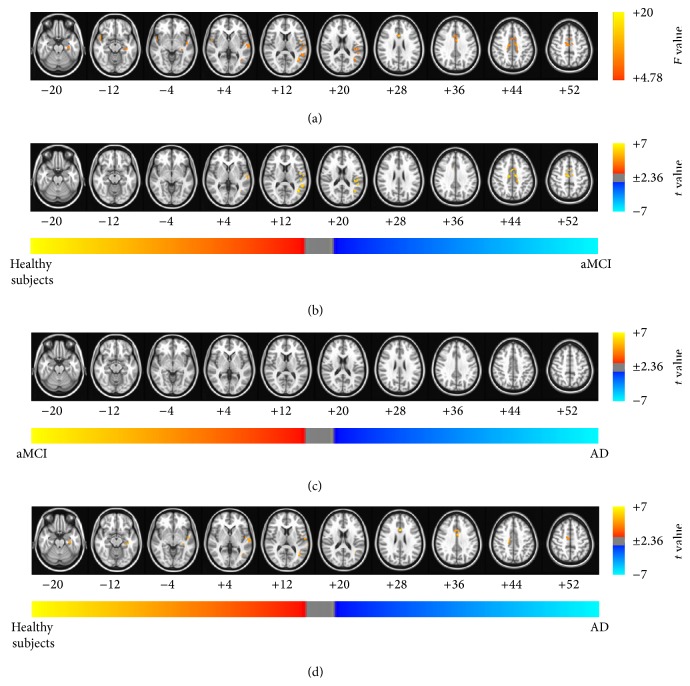
Brain regions exhibiting significant differences in the global brain connectivity (GBC) index. (a) Brain regions showed significant differences in the GBC index between healthy subjects and patients with aMCI and patients with AD (*P*_*α*_ < 0.05 (uncorrected *P* < 0.01, *F* > 4.78, 864 mm^3^, and AlphaSim corrected)). The results of the* post hoc* two-sample *t*-tests between pairs of the healthy subjects and patients with AD and aMCI were as follows: significant differences in the brain regions were found (b) in patients with aMCI compared with healthy subjects, (c) in patients with AD compared with patients with aMCI, and (d) in patients with AD compared with healthy subjects (*P*_*α*_ < 0.05). The images are oriented with the anterior side placed at the top and the left side placed to the right.

The results of the ANCOVA using age, sex, and education as covariates showed significant differences between the patients with aMCI and AD and healthy subjects (see Figures 2(a), 3(a), 4(a), and 5(a) and Tables 2–5 for details). Then, as shown in Figures 2(b)–2(d), 3(b)–3(d), 4(b)–4(d), and 5(b)–5(d) and Tables 6, 7, 8, and 9, we performed* post hoc* two-sample *t*-tests between pairs of groups. The ReHo, ALFF, fALFF, and GBC approaches showed that regions of the brain had decreased indices in patients with aMCI and AD compared with the healthy subjects. In particular, all RS FMRI approaches showed that the functional characteristics in the left parahippocampal gyrus were decreased in AD patients compared with healthy subjects. Therewith, significant group differences of the ReHo index were found in the middle temporal gyrus, ACC, postcentral gyrus, insula, precuneus, middle occipital gyrus, inferior parietal lobule, PCC, cingulate gyrus, and inferior frontal gyrus (*P*_*α*_ < 0.05; AlphaSim corrected, uncorrected *P* < 0.01 at a cluster size of at least 108 voxels; see Figure 2(a) and Table 2 for a detailed list of the regions). And significant group differences in the ALFF were found in superior temporal gyrus, medial frontal gyrus, parahippocampal gyrus, insula, superior frontal gyrus, caudate, and superior temporal gyrus (see Figure 3(a) and Table 3 for a detailed list of the regions). On the other hand, significant group differences in the fALFF were found in inferior parietal lobule, PCC, fusiform gyrus, middle frontal gyrus, precuneus, precentral gyrus, inferior frontal gyrus, middle temporal gyrus, parahippocampal gyrus, and cuneus (see Figure 4(a) and Table 4 for a detailed list of the regions). Significant group differences in the GBC index were found in the ACC, superior temporal gyrus, postcentral gyrus, parahippocampal gyrus, and cingulate gyrus (see Figure 5(a) and Table 5 for a detailed list of the regions).

## 4. Discussion

Here, we showed the functional alterations of the patients with aMCI and AD by applying several different RS FMRI techniques (ReHo, ALFF, fALFF, and GBC) to the data for healthy subjects and the data for patients with aMCI and patients with AD. In addition, these data also showed significant differences between healthy subjects and patients with aMCI and AD using the sICA reported in previous studies [19]. Although previous study showed differences between normal control and patients with MCI and AD with some similar method [32], this study was the first study of the whole brain voxel-based analysis. In conclusion, we showed that the results of using multiple approaches, excluding seed-based approaches, in RS FMRI analysis were useful for meta-analysis using the same data.

According to the various RS FMRI approaches, the patterns of functional alteration in patients with aMCI and AD were slightly different. Nevertheless, patients with aMCI and AD had significantly decreased functional characteristics compared with normal aging subjects for all approaches. Our major findings were as follows: (1) patients with aMCI and AD had decreased functional patterns compared with healthy subjects for all approaches. The ReHo, ALFF, fALFF, and GBC approaches showed that regions of the brain had decreased indices in patients with aMCI and AD compared with the healthy subjects. In particular, all RS FMRI approaches showed that the functional characteristics in the left parahippocampal gyrus were decreased in AD patients compared with healthy subjects, and (2) the ALFF and fALFF approaches showed that the indices in the posterior cingulate cortex (PCC), parahippocampal gyrus, middle temporal gyrus, and left inferior parietal lobule decreased significantly in the patients with AD compared with the patients with aMCI. The other methods did not show any differences between the patients with AD and aMCI. Taken together with the findings of our previous study, ALFF, fALFF, and sICA were found to be more sensitive methods than the other RS FMRI approaches in patients with aMCI and AD. These major findings strongly encourage meta-analysis in patients with aMCI and AD with RS FMRI.

The mean images of ReHo, ALFF, fALFF, and GBC for the three groups were very similar to those of the human DMN reported in previous studies [6, 33]. A previous study demonstrated that the ReHo maps showed the existence of the DMN prominently and consistently during the resting and conscious states [34]. The DMN also had significantly higher ALFF and fALFF during the resting state than the other brain areas [9–11, 29]. In addition, the GBC values mainly occurred in the DMN and CCN. A previous study showed that high GBC was found in both the CCN and DMN [17]. Therefore, the results of all four approaches were highly related to the DMN.

The regions of significant group differences from some of the different approaches were consistent with previous studies in patients with aMCI and AD [4, 7, 8, 12, 13, 18]. Interestingly, the* post hoc* two-sample *t*-tests between pairs of groups showed that the functional characteristics of all RS FMRI approaches in the left parahippocampal gyrus were decreased in AD patients compared with the healthy subjects. A previous study showed that there were structural changes in the left parahippocampal gyrus [35, 36] and reduced functional connectivity in the left parahippocampal gyrus [19]. The ReHo approach provides information about the intraregional functional characteristics, and the ALFF/fALFF approaches provide information about the oscillating brain activity. In addition, the GBC index provides information about the synchronization among remote areas. Therefore, from the perspective of both the intra- and interregional functional features, the functional characteristics in the left parahippocampal gyrus were decreased in patients with AD. In conclusion, the changes identified in the functional characteristics of the left parahippocampal gyrus provide a potential diagnosis of AD, regardless of the approach used to perform RS FMRI analysis.

Despite the consistency in the differences found between three groups by the four different methods, some inconsistency was exhibited because of the differences between these methods. The group differences observed using the ALFF approach were larger than those of the other approaches. Previous studies have shown that the ALFF method is more prone to noise from physiological sources, particularly near the ventricles and large blood vessels [10, 11]. Therefore, although we performed physiological noise removal with PESTICA, the results of the ALFF approach might still have been affected by noise. The fALFF approach was used to overcome this disadvantage and suppressed the group differences. The patterns of group differences observed using the fALFF approach were similar to those seen using the ReHo method. However, the fALFF approach showed differences between patients with AD and aMCI in the parahippocampal gyrus, cuneus, and middle temporal gyrus, whereas the ReHo approach did not show any difference between patients with AD and aMCI. The ALFF and fALFF approaches showed that the index in the PCC, parahippocampal gyrus, MTG, and left IPL was significantly decreased in the patients with AD compared with the patients with aMCI. The other methods did not show these differences between the patients with AD and aMCI.

Several additional issues need to be addressed. First, the number of subjects in this study (*n* = 133) was greater than those in previous studies. Therefore, this study might have greater statistical power than previous studies, and this might have caused different results to be obtained. However, patients with aMCI and AD displayed significantly decreased functional characteristics with every analysis approach, in agreement with previous studies. Second, when we performed analysis using the ALFF and fALFF approaches, we restricted the frequency band (0.009–0.08 Hz) to enable comparison with the other methods. A previous study suggested the patterns of ALFF and fALFF obtained patients with aMCI were sensitive to the choice of frequency band [15]. The ALFF and fALFF abnormalities were greater in the slow-5 band (0.01–0.027 Hz) than in the slow-4 band (0.027–0.073 Hz). Therefore, a study using various frequency bands is required for further analysis of alterations to the functional characteristics in patients with aMCI and AD. Third, our results might relate to the possible confounding interference of gray matter loss. Although the analyses of functional differences with controlling gray matter losses are important, there is a need to overcome some issues about notable resolution differences between EPI and T1 image. With the improved technical method, a further study is needed to examine relationship between functional connectivity and gray matter density.

## 5. Conclusions

Our study demonstrated differences in the functional characteristics of patients with aMCI and AD compared with healthy subjects using multimethod analysis. The patterns of functional alteration in patients with aMCI and AD were slightly different depending on the RS FMRI approach used. Nevertheless, patients with aMCI and AD had consistently decreased functional characteristics compared with healthy subjects, regardless of the approach used. All RS FMRI approaches showed that the functional characteristics in the left parahippocampal gyrus were decreased in AD patients compared with healthy subjects. The ALFF and fALFF approaches both showed that the index decreased significantly in the patients with AD compared with the patients with aMCI, whereas the other methods did not show such differences. Therefore, the ALFF, fALFF, and sICA techniques provided more sensitive measurements than the other RS FMRI approaches in patients with aMCI and AD. These major findings strongly encourage meta-analysis in patients with aMCI and AD with RS FMRI.

## Figures and Tables

**Table 1:** Demographic and clinical findings of healthy subjects and patients with amnestic mild cognitive impairment (aMCI) or Alzheimer's disease (AD).

	Healthy subjects	aMCI	AD	*P* value
Number of subjects	62	34	37	
MMSE score	28.6 ± 1.9	27.1 ± 2.1	16.8 ± 6.9	*P* < 0.0001
Age	68.5 ± 8.0	68.4 ± 7.9	72.8 ± 8.2	*P* < 0.03
Sex (M/F)	17/45	18/16	10/27	*P* < 0.03
Education	10.9 ± 5.2	11.5 ± 5.2	10.9 ± 5.3	*P* > 0.25

Data for age, education, and MMSE (mini-mental state examination) score: mean ± SD; M, male; F, female. The *P* value was obtained by one-way ANOVA and chi-square test.

**Table 2:** Brain regions with significant differences in regional homogeneity index between healthy subjects and patients with aMCI or AD.

Brain regions	R/L	Coordinates * *(mm)			Peak *F* values	Voxels
		*x*	*y*	*z*		
Healthy subjects versus aMCI versus AD						
Middle temporal gyrus	L	−40	−60	8	19.59	3286
Middle temporal gyrus	R	44	−58	4	16.07	848
ACC	L	−2	16	28	23.40	736
Middle temporal gyrus	R	32	−68	30	15.98	690
Postcentral gyrus	L	−36	−30	42	16.36	469
Postcentral gyrus	R	38	−32	56	15.62	301
Insula	R	38	16	6	15.86	272
ACC	R	14	28	28	16.29	224
Precuneus	R	2	−78	28	11.92	212
Middle occipital gyrus	R	34	−76	−6	12.02	186
IPL	L	−58	−26	28	14.60	171
Postcentral gyrus	R	52	−18	32	12.40	167
PCC	R	22	−58	18	16.72	162
Precuneus	L	−18	−68	16	13.20	160
Cingulate gyrus	L	−4	−34	30	11.61	160
Inferior frontal gyrus	L	−26	6	6	15.43	144

ACC: anterior cingulate cortex, IPL: inferior parietal gyrus, and PCC: posterior cingulate cortex. Threshold: corrected *P*_*α*_ < 0.05 (uncorrected individual voxel height threshold of *P* < 0.01, *F* > 4.776 with a minimum cluster size of 864 mm^3^).

**Table 3:** Brain regions with significant differences in amplitude of low-frequency fluctuations (ALFF) index between healthy subjects and patients with aMCI or AD.

Brain regions	R/L	Coordinates * *(mm)			Peak *F* values	Voxels
		*x*	*y*	*z*		
Healthy subjects versus aMCI versus AD						
Superior temporal gyrus	R	48	4	2	24.71	20320
Medial frontal gyrus	L	0	54	8	21.73	1412
Cerebellum	L	−24	−76	−50	20.83	1019
Parahippocampal gyrus	R	14	−6	−16	19.93	586
Cerebellum	R	20	−86	−40	13.57	573
Parahippocampal gyrus	R	16	−36	0	19.95	491
Middle frontal gyrus	R	20	30	42	9.51	279
Parahippocampal gyrus	R	36	−40	−4	14.35	270
Insula	R	24	28	10	17.84	258
Superior frontal gyrus	L	−18	34	44	9.96	230
Caudate	R	10	12	10	24.43	184
Superior frontal gyrus	L	−50	16	−32	12.75	166
Middle frontal gyrus	L	−24	−4	48	10.47	154

Threshold: corrected *P*_*α*_ < 0.05 (uncorrected individual voxel height threshold of *P* < 0.01, *F* > 4.776 with a minimum cluster size of 864 mm^3^).

**Table 4:** Brain regions with significant differences in fractional ALFF (fALFF) index between healthy subjects and patients with aMCI or AD.

Brain regions	R/L	Coordinates * * (mm)			Peak *F* values	Voxels
		*x*	*y*	*z*		
Healthy subjects versus aMCI versus AD						
Inferior parietal lobule	L	−54	−56	48	15.82	1654
PCC	L	−8	−60	12	13.17	743
Fusiform gyrus	R	38	−40	−8	16.58	461
Cerebellum	L	−24	−12	−36	12.98	422
Middle frontal gyrus	L	−40	38	18	13.38	416
Inferior parietal lobule	R	50	−40	46	9.66	246
Precuneus	R	14	−68	40	9.14	205
Middle frontal gyrus	R	10	58	22	12.49	201
Precentral gyrus	L	−52	2	8	13.33	200
Postcentral gyrus	L	−48	−30	36	12.26	195
Inferior frontal gyrus	R	48	22	16	12.23	167
Middle temporal gyrus	R	58	6	−22	13.34	164
Superior temporal gyrus	R	50	−42	16	8.70	163
Parahippocampal gyrus	R	34	−12	−28	10.76	146
Cuneus	R	14	−68	6	12.11	140
Medial frontal gyrus	L	−2	52	−4	9.09	134

PCC: posterior cingulate cortex. Threshold: corrected *P*_*α*_ < 0.05 (uncorrected individual voxel height threshold of *P* < 0.01, *F* > 4.776 with a minimum cluster size of 864 mm^3^).

**Table 5:** Brain regions with significant differences in global brain connectivity (GBC) index between healthy subjects and patients with aMCI or AD.

Brain regions	R/L	Coordinates * *(mm)			Peak *F* values	Voxels
		*x*	*y*	*z*		
Healthy subjects versus aMCI versus AD						
ACC	L	−2	12	28	15.36	763
Superior temporal gyrus	L	−68	−24	6	11.24	282
Cerebellum	L	−28	−46	−48	10.10	253
Postcentral gyrus	L	−20	−52	66	8.41	208
Superior temporal gyrus	L	−38	−58	12	16.27	170
Superior temporal gyrus	L	−50	−42	14	9.37	159
Parahippocampal gyrus	L	−38	−26	−12	8.74	153
Cingulate gyrus	L	−12	−14	40	14.49	145
Transverse temporal gyrus	L	−50	−24	12	7.29	143
Superior temporal gyrus	R	46	8	−16	12.21	127

ACC: anterior cingulate cortex. Threshold: corrected P_α_ < 0.05 (uncorrected individual voxel height threshold of P < 0.01, F > 4.776 with a minimum cluster size of 864 mm^3^).

**Table 6:** Results of *post hoc* two-sample *t*-tests between every pair of the healthy subjects and patients with AD and aMCI groups in ReHo approach.

Brain regions	R/L	Coordinates * *(mm)			Peak *t* value	Voxels
		*x*	*y*	*z*		
Healthy subject versus aMCI						
Superior parietal lobule	L	−18	−76	56	4.27	531
Cingulate gyrus	L	−4	−4	48	5.12	381
Precuneus	R	26	−62	50	5.59	284
Middle occipital gyrus	L	−38	−66	−12	5.39	279
Precentral gyrus	L	−40	−18	42	4.88	270
Superior temporal gyrus	L	−50	−2	2	5.25	266
Superior temporal gyrus	L	−54	−42	16	4.83	250
Precentral gyrus	R	40	−24	60	4.98	235
Middle occipital gyrus	L	−52	−72	2	5.07	220
Cingulate gyrus	R	8	24	38	5.19	184
Middle temporal gyrus	R	34	−70	26	4.27	177
Postcentral gyrus	R	52	−18	32	4.94	149
Insula	R	38	16	6	4.63	146
aMCI versus AD No result						
Healthy subject versus AD						
Middle temporal gyrus	L	−40	−60	8	6.22	1460
Middle temporal gyrus	R	44	−58	4	5.44	735
ACC	L	−2	16	28	6.49	551
Middle temporal gyrus	L	−50	−20	−8	5.31	399
Middle temporal gyrus	R	32	−68	30	5.21	329
Precentral gyrus	R	36	−24	54	4.97	197
Superior parietal lobule	L	−34	−56	54	4.58	184
Insula	R	30	22	12	4.49	178
Postcentral gyrus	L	−50	−20	40	4.89	175
Precuneus	R	2	−78	28	4.76	165
Parahippocampal gyrus	L	−32	−10	−20	5.01	164
Cingulate gyrus	L	−4	−34	30	4.80	160
Precuneus	L	−18	−68	16	5.06	159
PCC	R	22	−58	18	5.78	155
Middle occipital gyrus	R	36	−86	−6	4.74	149
Cerebellum	L	−26	28	4	5.28	125

ACC: anterior cingulate cortex, PCC: posterior cingulate gyrus. Positive values: healthy subjects > aMCI, aMCI > AD, and healthy subjects > AD. Negative values: aMCI > healthy subjects, AD > aMCI, and AD > healthy subjects. Threshold: corrected *P*_*α*_ < 0.05.

**Table 7:** Results of *post hoc* two-sample *t*-tests between each pair of the healthy subjects and patients with AD and aMCI groups in ALFF approach.

Brain regions	R/L	Coordinates * * (mm)			Peak *t* value	Voxels
		*x*	*y*	*z*		
Healthy subject versus aMCI						
Inferior parietal lobule	R	56	−46	24	3.87	729
Supramarginal gyrus	L	−52	−50	24	3.98	585
Middle temporal gyrus	L	−30	−68	26	4.94	428
Middle occipital gyrus	R	34	−80	8	4.15	413
Postcentral gyrus	R	52	−8	16	3.46	353
Parahippocampal gyrus	L	−24	−40	−12	3.44	335
Superior temporal gyrus	R	50	6	0	4.59	222
Precuneus	R	2	−68	18	3.49	221
Middle temporal gyrus	L	−44	−62	4	3.78	217
Postcentral gyrus	R	52	−18	32	3.73	217
Medial frontal gyrus	R	4	46	42	3.60	202
Precuneus	L	0	−50	32	3.69	188
Precuneus	L	−16	−76	42	3.43	128
aMCI versus AD						
Precuneus	L	0	−70	46	4.19	654
Cerebellum	L	−22	−84	−44	4.75	594
Cerebellum	R	16	−84	−38	3.73	350
Parahippocampal gyrus	L	−26	−8	−18	4.34	345
Middle temporal gyrus	L	−48	−66	22	4.08	306
Superior temporal gyrus	L	−40	18	−28	4.30	185
Healthy subject versus AD						
Superior temporal gyrus	R	48	4	2	3.13	20151
Medial frontal gyrus	L	0	54	8	6.54	1411
Cerebellum	L	−24	−76	−50	6.22	957
Parahippocampal gyrus	R	14	−6	−16	6.30	575
Cerebellum	R	20	−86	−40	5.12	564
Parahippocampal gyrus	R	14	−36	2	6.15	490
Medial frontal gyrus	R	20	30	42	4.17	278
Parahippocampal gyrus	R	36	−40	−4	5.11	270
Lentiform Nucleus	R	24	6	20	−5.82	258
Superior frontal gyrus	L	−18	3	44	4.36	230
Caudate	R	10	12	10	6.78	184
Superior temporal gyrus	L	−50	16	−32	4.73	164
Medial frontal gyrus	L	−26	−6	48	4.41	153

Positive values: healthy subjects > aMCI, aMCI > AD, and healthy subjects > AD; Negative values: aMCI > healthy subjects, AD > aMCI, and AD > healthy subjects; Threshold: corrected *P*_*α*_ < 0.05.

**Table 8:** Results of *post hoc* two-sample *t*-tests between every pair of the healthy subjects and patients with AD and aMCI groups in fALFF approach.

Brain regions	R/L	Coordinates * * (mm)			Peak *t* value	Voxels
		*x*	*y*	*z*		
Healthy subject versus aMCI						
Middle temporal gyrus	L	−30	−72	24	3.89	256
Superior temporal gyrus	L	−56	8	0	4.32	143
Inferior parietal lobule	L	−42	−58	38	4.14	136
Inferior parietal lobule	L	−50	−46	22	4.14	131
aMCI versus AD						
Parahippocampal gyrus	R	42	−30	−16	4.61	249
Cuneus	R	0	−72	6	4.23	173
Cerebellum	L	−26	−12	−36	4.48	163
Parahippocampal gyrus	R	34	−12	−28	4.60	135
Middle temporal gyrus	R	60	4	−22	4.04	131
Healthy subject versus AD						
Inferior parietal lobule	R	−48	−62	42	5.37	2324
PCC	L	−8	−62	12	4.92	832
Fusiform gyrus	R	38	−40	−8	5.75	504
Middle frontal gyrus	L	−40	38	18	5.10	409
Middle temporal gyrus	L	−48	10	−32	4.79	399
Inferior parietal lobule	R	50	−40	46	4.33	242
Precuneus	R	26	−60	22	4.17	205
Medial frontal gyrus	R	10	58	22	4.95	198
Postcentral gyrus	L	−58	−26	38	4.75	191
Inferior frontal gyrus	R	48	22	16	4.85	167
Superior temporal gyrus	R	60	−46	14	3.92	163
Middle frontal gyrus	R	58	6	−22	4.97	147
Cuneus	R	14	−68	6	4.84	140
Postcentral gyrus	L	−52	2	10	4.65	136
Medial frontal gyrus	L	−2	52	−5	4.04	125

PCC: posterior cingulated cortex. Positive values: healthy subjects > aMCI, aMCI > AD, and healthy subjects > AD. Negative values: aMCI > healthy subjects, AD > aMCI, and AD > healthy subjects. Threshold: corrected *P*_*α*_ < 0.05.

**Table 9:** Results of *post hoc* two-sample *t*-tests between every pair of the healthy subjects and patients with AD and aMCI groups in GBC approach.

Brain regions	R/L	Coordinates * *(mm)			Peak t value	Voxels
		*x*	*y*	*z*		
Healthy subject versus aMCI						
Superior frontal gyrus	R	2	2	58	4.26	525
Postcentral gyrus	L	−20	−52	66	4.04	207
Precentral gyrus	L	−48	−4	12	3.59	162
Middle temporal gyrus	L	−40	−58	10	5.88	158
Superior temporal gyrus	L	−50	−42	14	4.19	154
Transverse temporal gyrus	L	−50	−24	12	3.77	143
Cingulate gyrus	L	−12	−14	40	4.06	136
aMCI versus AD						
Cerebellum	L	−28	−48	−52	3.65	141
Healthy subject versus AD						
Superior temporal gyrus	L	−68	−24	6	4.36	258
Cerebellum	L	−28	−46	−48	4.48	246
ACC	L	−2	12	28	5.53	241
Cingulate gyrus	R	16	−20	40	5.09	165
Parahippocampal gyrus,	L	−36	−28	−12	4.07	153
Superior temporal gyrus	L	−38	−58	12	130	130

ACC: anterior cingulate gyrus. Positive values: healthy subjects > aMCI, aMCI > AD, and healthy subjects > AD. Negative values: aMCI > healthy subjects, AD > aMCI, and AD > healthy subjects. Threshold: corrected *P*_*α*_ < 0.05.
